# Egr1 confers protection against acetaminophen‑induced hepatotoxicity via transcriptional upregulating of Acaa2

**DOI:** 10.7150/ijbs.71781

**Published:** 2022-05-29

**Authors:** Xiaohong Lei, Qingling Xu, Chunmin Li, Baolin Niu, Yanan Ming, Jing Li, Yingyue Tang, Xiaoyun Li, Jieting Tang, Jing Wu, Yi Ju, Lvfeng Yao, Bin Wang, Qi Miao, Wei Zhong, Yang Zhi, Lirong Xu, Chaojun Li, Xiaobo Li, Yimin Mao

**Affiliations:** 1 Division of Gastroenterology and Hepatology; Shanghai Institute of Digestive Disease; NHC Key Laboratory of Digestive Diseases; Renji Hospital, School of Medicine, Shanghai Jiao Tong University, Shanghai, 200001, China.; 2 Department of Physiology and Pathophysiology, School of Basic Medical Sciences, Fudan University, Shanghai, 200032, China; 3 Department of Hepatology, Mengchao Hepatobiliary Hospital of Fujian Medical University, Fuzhou, Fujian, 350025, China; 4 Department of Gastroenterology and Hepatology, Shanghai General Hospital, Shanghai Jiao Tong University School of Medicine, Shanghai, 200080, China; 5 Institute of Pathology, University Hospital Heidelberg, Heidelberg, 69120, Germany; 6 Model Animal Research Centre (MARC), Medical School of Nanjing University, National Resource Centre for Mutant Mice, Nanjing, 210093, China; 7 Department of Pathology, School of Basic Medical Sciences, Fujian Medical University, Fuzhou, Fujian, 350122, China; 8 Department of Pathology, School of Basic Medical Science, Shanghai University of Traditional Chinese Medicine, Shanghai, 201203, China; 9 State Key Laboratory of Reproductive Medicine, Centre for Global Health, School of Public Health, Nanjing Medical University, Nanjing, 211100, China

**Keywords:** Acetaminophen-induced liver injury, Drug-induced liver injury, Early growth response 1, fatty acid β-oxidation

## Abstract

***Background***: Acetaminophen (APAP)-induced liver injury (AILI) is a common cause of drug-induced liver injury (DILI). The mechanism underlying protection in AILI or DILI remains to be elucidated, and the role of early growth response 1 (Egr1) in AILI and potential mechanisms remain to be known.

***Methods***: The role of Egr1 was studied both *in vivo* and *in vitro*. Liver-specific *Egr1*-knockout (*Egr1*^LKO^) mice and those overexpressing Egr1 via tail vein injection of Egr1-expressing adenovirus (Ad-Egr1) were utilized with AILI. Chromatin immunoprecipitation-sequencing, RNA-sequencing, seahorse XF analysis, and targeted fatty acid analysis were performed. EGR1 levels were also studied in liver tissues and serum samples from AILI/DILI patients.

***Results:*** In this study, we have demonstrated that Egr1 was upregulated in AILI models *in vivo* and *in vitro*. liver-specific *Egr1* knockout aggravated AILI; however, Ad-Egr1 treatment ameliorated this. Mechanistically, Egr1 deficiency inhibited, whereas overexpression promoted, mitochondrial respiratory function and fatty acid β-oxidation (FAO) activity in AILI. Egr1 transcriptionally upregulated FAO-related genes in hepatocytes. Notably, the knockdown of *acetyl-coenzyme A acyltransferase 2* (*Acaa2*), a key gene involved in FAO, diminished this protective effect of Egr1. Clinically, EGR1 was markedly increased in liver tissues from AILI patients. Interestingly, EGR1 levels of liver tissues and serum samples were also obviously higher in idiosyncratic DILI patients.

***Conclusions:*** Egr1 confers adaptive protection in AILI, mediated via the transcriptional upregulation of Acaa2, which improves mitochondrial FAO, and might be a potential biomarker and novel therapeutic target for AILI.

## Introduction

Drug-induced liver injury (DILI) is the major cause for the withdrawal of approved drugs from the market, and can cause liver failure or even fatality 1-3. Acetaminophen (APAP) is widely used for its analgesic and antipyretic properties. However, the accidental and intentional overdose of APAP is the primary cause of DILI and acute liver failure 4, 5.

The pathogenesis of APAP induced liver injury (AILI) is complicated and not fully understood 6-11. The reported pathogenesis of APAP induced hepatotoxicity is initiated by N-acetyl-p-benzoquinone imine (NAPQI), which is generated by cytochrome P450 (CYP) enzymes, particularly CYP2E1, and detoxified via conjugation with hepatic glutathione (GSH) 12-14. Mitochondrial dysfunction and oxidative stress are central to the mechanisms of APAP-induced hepatotoxicity 15-18. Therefore, suppressing oxidative stress and protecting mitochondrial function may serve as efficient potential strategies to alleviate APAP hepatotoxicity.

The product of the *early growth response 1* (*Egr1*) gene is a zinc finger transcription factor with three zinc finger motifs in the DNA-binding domain 19.* Egr1*, as an immediate early gene, has been indicated for its rapid and transient induction in response to various environmental stimuli 20. The Egr1 protein binds to the GC-rich 5′-GCGGGGGCG-3′ sequence for regulating the transcription of target genes involved in cellular growth, proliferation, differentiation, or cell death 19, 20. Currently, the role of Egr1 in liver diseases remains controversial. Many studies demonstrated that Egr1 could activate the transforming growth factor (TGF)-β1/Smad signaling pathway, or upregulate the levels of inflammatory signaling pathway to accelerate the development of liver injury 21-23. However, Pritchard et al. reported that Egr1 up-regulated the expression of TNFα and other hepatoprotective molecules, and prompted the passage of cells from the G0 to G1 phase and induced G1/S phase transition, which contributed to alleviating CCl4-induced liver injury 24, 25. Two studies have reported the effect of Egr1 on APAP-induced hepatotoxicity; however, the results were inconsistent 26, 27. Moreover, Egr1 could transcriptionally regulate many genes involved in metabolic homeostasis, such as glucose metabolism 28, 29, lipid and cholesterol metabolism 30 and fatty acid metabolism 31. But so far, the association between Egr1 and metabolic disorders of AILI has not been previously explored.

Taken together, the hypothesis of our study was that Egr1 might play an important role in AILI. In this study, we focus on the functional role and underlying mechanism of Egr1 in acute AILI. The data reveal that Egr1 confers adaptive protection in AILI, mediated via the transcriptional upregulation of Acaa2, which improves mitochondrial FAO.

## Materials and Methods

### Collection of samples from patients and controls

Patients were enrolled from the Renji Hospital in Shanghai, China between October 2017 and February 2021. And patients in this study were from two separate DILI cohorts. The study protocol was approved by the Ethics Committee of Renji Hospital, School of Medicine, Shanghai Jiaotong University. All patients met the following inclusion criteria: (ⅰ) male and female patients > 18 years of age; (ii) met international consensus criteria for DILI at the first set of liver biochemistry available in relation to the clinical event 32, 33; (ⅲ) patients with RUCAM scores ≥ 6, or patients with RUCAM scores < 6 were further judged by three hepatologists with DILI expertise as “probable” DILI; (ⅳ) other etiologies of liver injury were excluded; (ⅴ) all participants provided written informed consent. In total, 45 DILI cases (liver tissues from 24 patients, serum samples from another 21 patients) and 33 healthy controls (liver tissues from 16 liver transplant donors, serum samples from another 17 healthy volunteers) were enrolled in this study. The clinical data pertaining to these 45 patients were provided in Table S1 and S2.

### Animals

C57BL/6J mice, aged 6-8 weeks, were purchased from the Experimental Animal Center of Shanghai SLAC (Shanghai, China). The mice with liver-specific *Egr1* knockout (*Egr1*^LKO^) were kindly provided by Professor Chaojun Li (Nanjing University Medical University, China) by crossing Alb-Cre transgenic mice with homozygous floxed *Egr1* (*Egr1*^fl/fl^) mice (on a C57BL/6J background) 34, 35. The littermate *Egr1*^fl/fl^ mice were used as the wild-type controls. The mice were fed on a normal diet and had *ad libitum* access to water. The animals were housed in specific pathogen-free conditions at 24 ± 2°C under a 12 h light-dark cycle, at a relative humidity of 50 ± 5%. All the animal studies were approved by the Institutional Animal Care and Use Committee of Renji Hospital, School of Medicine, Shanghai Jiao Tong University.

### Animal studies

All the mice were fasted overnight for approximately 12 h before the intraperitoneal (i.p.) injection of APAP (Sigma Aldrich, USA) at a dose of 750 mg/kg (body weight), 300 mg/kg (body weight), or warm saline (0.9% NaCl; 37°C), as previously described 36. The mice were euthanized after 1, 3, 6, 12, or 24 h of treatment with APAP or saline. Blood samples were subsequently collected and centrifuged at 3,000 × g for 15 min for obtaining the serum. The serum samples and liver tissues were stored at -80°C.

### Adenovirus production and infection

The Egr1 (Ad-Egr1) and control (Ad-GFP) adenoviruses were purchased from Hanbio Biotechnology Co., Ltd. (Shanghai, China). Ad-Egr1 or Ad-GFP was injected via the lateral tail vein of each mouse at a dose of 5 × 10^8^ viral titer. The mice were fasted overnight on the second day after the injection. On the third day, the mice received i.p. injection of APAP (300 mg/kg) and subsequently euthanized after 12 h or 24 h. For the *in vitro* study, AML12 and Hepa1-6 cells were incubated with Ad-Egr1 or Ad-GFP for 48 h, followed by treatment with APAP at the indicated time intervals.

### Small interfering RNA (siRNA)-mediated knockdown of acetyl-Coenzyme A acyltransferase 2 (Acaa2)

We used the Acaa2 siRNA (si-Acaa2) for inhibiting the expression of Acaa2. To this end, si-Acaa2 and the corresponding negative controls (si-CON) were designed from RiboBio (Guangzhou, China). The si-Acaa2 or si-CON was injected into 6-8-week-old C57BL/6J mice via the tail vein at an effective concentration of 20 mM. After 24 h, Ad-Egr1 or Ad-GFP was injected into the mice over a duration of 48 h, followed by the i.p. administration of APAP (300 mg/kg). The mice were sacrificed after 6 h of APAP administration. The *in vitro* transfections with si-Acaa2 and si-CON were performed using Lipofectamine 3000 (Invitrogen, USA). The sequence of si-Acaa2 was 5'-UGCUGAGACAGUGAUUGUATT-3'/5'-UACAAUCACUGUCUCAGCATT-3'.

### Biochemical assays and histopathological analyses

The serum levels of alanine aminotransferase (ALT), aspartate aminotransferase (AST), and lactate dehydrogenase (LDH) were analyzed using an automatic biochemical analyzer (Siemens ADVIA 1800, Siemens Healthcare Diagnostics, USA). The frozen and paraffinized liver tissues were stained with Oil Red O, hematoxylin and eosin (H&E), respectively. Terminal dUTP nick-end labeling (TUNEL) staining was performed using *in situ* cell detection kit (Roche, Switzerland).

### Isolation of primary mouse hepatocytes (PMHs)

Primary hepatocytes were isolated from mice using collagenase IV perfusion, as previously described 37. The digested liver tissues were filtered through a 70 μm nylon mesh. The PMHs were collected after centrifugation at 50 × g for 5 min. The cells were washed twice and resuspended in Dulbecco's modified Eagle's medium (DMEM, Biological Industries, Israel) supplemented with 10% fetal bovine serum. The cells were subsequently incubated 4 h with 5% CO_2_ at 37°C for allowing adhesion.

### Immunohistochemistry staining

The formalin-fixed, paraffin-embedded sections of liver tissues were incubated overnight with anti-rabbit Egr1 antibody (Cell Signaling Technology Cat# 4154) at 4°C. The tissues were subsequently incubated with a horseradish peroxidase-conjugated anti-rabbit secondary antibody at 37°C for 1 h. The tissue sections were visualized using DAB and counterstained with hematoxylin. The immunohistochemistry staining was scored by three independent pathologists. The staining intensity was scored as follows: 0, no staining; 1, weak staining; 2, intermediate staining; and 3, strong staining. The positive rate score was determined as follows: 0, 0-4% of the cells stained positive; 1, 5-25% of the cells stained positive; 2, 26-50% of the cells stained positive; 3, 51-75% of the cells stained positive; and 4, 76-100% of the cells stained positive. The histological score (H-score) was multiplied by the intensity and positive rate scores.

### Immunofluorescence staining

The cells were seeded onto slides covered with 24-well plates and treated as indicated. The slides were incubated overnight with anti-rabbit Egr1 antibody (Cell Signaling Technology Cat# 4154) at 4°C, followed by incubation with a fluorescent secondary antibody at room temperature for 1 h. The nuclei were stained with DAPI for 15 min at room temperature. The images were captured using a fluorescence microscope (Zeiss, Gruppe, Germany).

### Measurement of GSH

The total GSH in 100 mg liver tissue was measured using a GSH Quantification Kit (Dojindo Molecular Technologies, Japan), according to the manufacturer's instructions.

### Quantitative real-time polymerase chain reaction (RT-qPCR) analysis

The total RNA was isolated using TRIzol reagent (Life Technologies, Thermo Fisher Scientific), according to the manufacturer's instructions. The cDNA was prepared using Prime Script RT reagent kit (Takara, Shiga, Japan), and qRT-PCR was performed using the SYBR Green PCR kit (Yeasen, China). The sequences of the primers used for the gene expression studies were listed in Table S3.

### Luciferase reporter assay

A fragment of the murine Acaa2 promoter from -1 bp to -1211 bp (PGL4 Acaa2-N) was inserted into the pGL4-Basic vector (Transheep, China). The AML12 cells were seeded in 24-well plates at a density of 1.5 × 10^3^ cells per well, 24 h before transfection. The cells were incubated with Ad-Egr1 or Ad-GFP for 24 h, and subsequently co-transfected with 0.5 µg of Acaa2 reporter vectors and 0.25 µg pRL vector (Renilla luciferase control reporter vector), by incubation with Lipofectamine 3000 (Invitrogen, USA) for 24 h. The luciferase activity was measured using a dual luciferase reporter assay system (Yeasen, China). The different truncated fragments of Acaa2 (from -1 bp to -927 bp, -1 bp to -720 bp, -46 bp to -1211 bp, -232 bp to -1211 bp, and -375 bp to -1211 bp) were cloned into the vectors. The fragment from -31 bp to -45 bp was mutated using the QuikChange II Site-Directed Mutagenesis Kit (Stratagene, La Jolla, CA, USA). The relative luciferase activity was finally determined by comparing the activity of the firefly luciferase with that of Renilla luciferase.

### Western blot analysis

The total protein was extracted from the liver tissues and cells using RIPA lysis buffer (Beyotime, China). The nuclear and cytoplasmic protein fractions were obtained from the liver tissues using a Nuclear Protein Extraction kit (Beyotime, China). The protein was quantified using the BCA protein assay (Thermo Scientific, USA), and protein was separated by SDS-PAGE and transferred to PVDF membranes (Millipore, USA). After blocking with 3% bovine serum albumin (BSA) in TBST for 90 min at room temperature, the membranes were incubated overnight with the primary antibodies against Egr1 (Santa Cruz Biotechnology, Inc. Cat# sc-101033), CYP2E1 (Proteintech Cat# 19937-1-AP), Acaa2 (Cohesion Biosciences Cat# CQA3736), HSP90 (Proteintech Cat# 13171-1-AP), GAPDH (Proteintech Cat# 60004-1-Ig), and Histone H3 (Cell Signaling Technology Cat# 9715) at 4°C. After incubation with horseradish peroxidase-labeled secondary antibodies for 1 h at room temperature, the protein signals were detected using a Pierce ECL western blotting Kit (Thermo Scientific, USA).

### Cell viability assay

The PMHs viability assay was performed using the CellTiter-Glo™ Luminescent Cell Viability Assay Kit (Promega, USA), according to the manufacturer's protocol. The PMHs were seeded in 96-well plates, following which the medium was replaced with DMEM, with or without 20 mM APAP, at different time intervals. The viability reagent was subsequently added to the PMHs and shaken for 2 min. The mixture was subsequently incubated for 10 min at room temperature, and cell viability was assessed using a Synergy™ LX Multi-Mode Microplate Reader (Biotek, USA). The viability of AML12 cells was measured using a Cell Counting Kit-8 assay (Dojindo Laboratories, Japan). Optical density was recorded at 450 nm.

### Seahorse XFe96 metabolic flux analysis

Mitochondrial function was determined using an XFe96 Seahorse Extracellular Flux Analyzer (Seahorse Bioscience, Billerica, MA, USA). The hepatocytes were plated on Seahorse XFe96 culture plates. Following 4 h attachment, the cells were treated with APAP (20 mM) for 3 h. Each of the wells in the plate were subsequently injected with the ATP synthase inhibitor, oligomycin (2 μM), the electron transport chain (ETC) accelerator, FCCP (1 μM), and a mitochondrial inhibitor, rotenone (1 μM)/antimycin (1 μM). The basal mitochondrial respiration was determined by subtracting the non-mitochondrial respiration from the basal cellular respiration, while the maximal respiratory capacity was calculated by subtracting the non-mitochondrial respiration from the maximal uncoupled respiration. For determining the exogenous fatty acid β-oxidation (FAO) flux, palmitate-BSA (PA-BSA, 0.175 mM) or BSA (0.03 mM) was added to the appropriate wells, followed by the injection of oligomycin (2 μM), FCCP (4 μM), and rotenone (1 μM)/antimycin (1 μM), and then measured using an XFe96 Seahorse Extracellular Flux Analyzer.

### Transmission electron microscopy

The PMHs were treated as indicated and fixed for 2 h with 2.5% glutaraldehyde/phosphate buffer, followed by secondary fixation using 1% osmium tetroxide. Following dehydration, embedding, and staining, images of the samples were acquired using a transmission electron microscope (JEOL, Japan).

### Measurement of the serum levels of CK18-M30, CK18-M65, EGR1 and liver levels of triglyceride (TG) by enzyme-linked immunosorbent assay (ELISA)

The serum levels of CK18-M30 and M65 in mice were quantified using an ELISA kit (LiankeBio, China). The serum levels of EGR1 in the human samples were quantified using an ELISA kit (Signalway Antibody, USA). The liver levels of TG in mice were quantified using an ELISA kit (Jiancheng Bio, Nanjing, China).

### RNA-sequencing (RNA-Seq) analysis

The total RNA from the liver tissues was extracted using the TRIzol reagent (Invitrogen, USA). The RNA-seq libraries were prepared using the NEBNext Ultra Directional RNA Library Prep kit for Illumina (New England Biolabs, USA), according to the manufacturer's instructions. The RNA-seq datasets were processed and analyzed as previously described 38. The differentially expressed genes (over 1.5-fold, P < 0.05) were analyzed using Deseq2. The gene ontology (GO) were analyzed using DAVID (http://david.abcc.ncifcrf.gov/home.jsp). The raw RNA-seq data were deposited in the NCBI SRA database under the accession number PRJNA744757.

### Chromatin immunoprecipitation-sequencing (ChIP-Seq) analysis

ChIP studies of the liver tissues were performed using the ChIP-IT High Sensitivity kit (Active Motif, USA), according to the manufacturer's protocol. The tissues were homogenized with a hand-held tissue homogenizer for 30 s at 30,000 rpm, followed by homogenization with 30 strokes of a tight-fitting pestle. The homogenized chromatin was then sheared using Active Motif EpiShearTM Probe Sonicator (Active Motif, USA) to obtain fragments of approximately 250-500 bp. The sheared chromatin (250 μg) was immunoprecipitated using primary antibodies against Egr1 (Cell Signaling Technology Cat# 4154) and IgG (Cell Signaling Technology Cat# 3900). Following overnight incubation, the ChIP-DNA was eluted and de-crosslinked for ChIP-seq analysis.

ChIP sequencing was performed by the Beijing Genomics Institute (Shenzhen, China), using Illumina HiSeq 2000. The raw sequenced reads were mapped to mouse genome51 (mm9) with bwa52 (ver. 0.6.2-r126). The regions that were enriched in the immunoprecipitated samples were identified using model-based analysis of ChIP-seq (MACS2 version 2.1.0) with a threshold of P < 0.05.

### Free fatty acid (FFA) analyses

For GC-MS-based FFA measurements, 5 × 10^6^ cells were collected and quickly frozen in liquid nitrogen before the extraction of metabolites in acetonitrile/isopropanol/water (3/3/2, v/v). The standard calibration solutions of 37 standard fatty acid methyl esters were prepared at 11 different concentration levels. The FFAs were separated on an Agilent HP-INNOWAX column (30 m × 0.25 mm) using helium as the carrier gas. The initial temperature of the oven was kept at 50°C for 3 min, and was programmed to increase at a rate of 10°C/min to 220°C, and maintained for 3 min. The temperature was finally increased at a rate of 15°C/min to 250°C, and maintained for 10 min. The peaks representing each metabolite were analyzed based on the peaks of the standard FFAs.

### Statistical analyses

All the experimental data were presented as the mean ± standard error of the mean (SEM). The two-parameter comparisons were performed using two-tailed Student's *t-tests*. The multiple-group analyses were performed using one-way analysis of variance (ANOVA) with Tukey's post hoc test. *P* < 0.05 was considered as statistically significant. All the statistical analyses were performed using GraphPad Prism software (version 8.0; San Diego, CA, USA).

## Results

### Egr1 was significantly upregulated in acute AILI models

**Fig 1:**
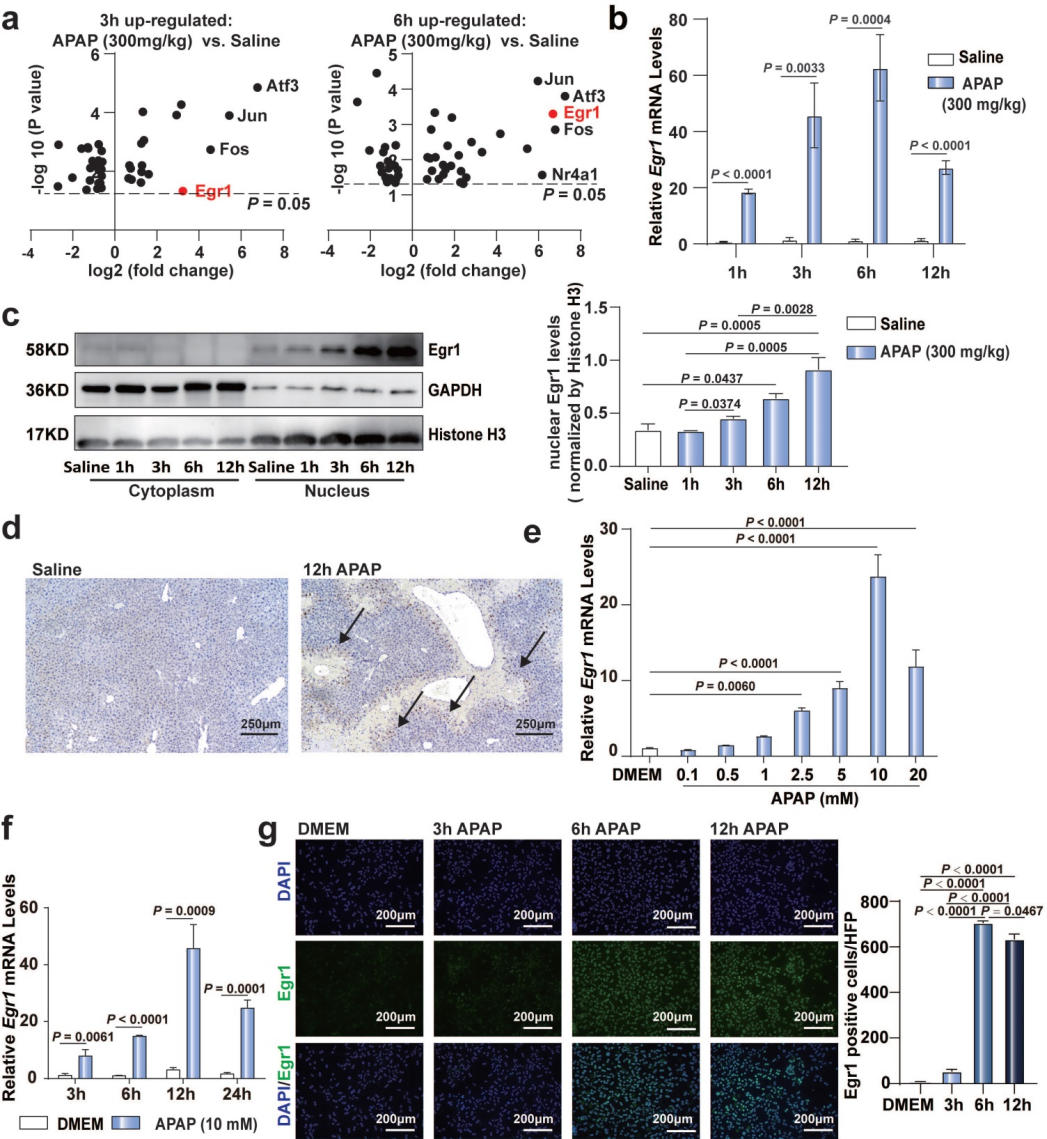
**Egr1 was significantly upregulated in acute AILI models. a.** Volcano plots of transcriptional factors from liver tissue RNA sequencing data of saline group, 3 h AILI group and 6 h AILI group (*P* < 0.05, n = 3 mice/group,* t* test). **b.** Relative *Egr1* mRNA levels of liver tissue in saline and APAP groups at 1, 3, 6, and 12 h (n = 6 mice/group,* t* test). **c.** Western blot analysis of Egr1 levels of cytoplasmic and nuclear protein in the liver tissue of saline and APAP groups at 1, 3, 6, and 12 h, followed by quantified protein levels (one-way ANOVA). **d.** Immunohistochemical staining images of Egr1 in the liver tissue of saline group and 300 mg/kg APAP groups at 12h (scale bar = 250 μm). Black arrows represent positive staining. **e.** Relative *Egr1* mRNA levels of PMHs treated with different doses of APAP (one-way ANOVA). **f.** Relative *Egr1* mRNA levels of PMHs treated with DMEM and 10 mM APAP at different time points (*t* test). **g.** Representative images of Egr1 fluorescence in PMHs treated with 10 mM APAP for 0, 3, 6, and 12 h (scale bar = 200 μm), followed by quantified the numbers of Egr1 positive cells per high-power field (HPF) (one-way ANOVA).

To confirm the significance of Egr1 in AILI models, we re-analyzed our previously published RNA sequencing data (PRJNA731100) 38. Of the over 40 transcriptional factors, the expression levels of *Egr1, Atf3*, *Jun*, and *Fos* were markedly upregulated in the liver tissues following challenge with 300 mg/kg APAP at 3 h and 6 h (Fig. 1a). To date, the role of Egr1 in acute AILI remains unclear. Next, we detected the expression of Egr1 in APAP-injured liver tissues and PMHs. The mRNA levels of *Egr1* in the liver tissues of mice after 1, 3, 6, and 12 h of 300 mg/kg APAP challenge were markedly increased (Fig. 1b). Egr1 was dominantly expressed in the nuclei of hepatocytes. The nuclear protein levels of Egr1 were significantly increased after 6 h and 12 h of APAP challenge (Fig. 1c). Meanwhile, immunohistochemical staining revealed that the Egr1 protein was located at the hepatocytic nuclei, at the boundary of the necrotic area in the liver tissues of mice treated with APAP, but not in the liver tissues of mice treated with saline (Fig. 1d). APAP gradually increased the mRNA levels of *Egr1* in the PMHs in a dose- and time-dependent manner, showing a maximal increase at 10 mM and 12 h (Fig. 1e and 1f). Immunofluorescent staining revealed that Egr1 was located at the nuclei of PMHs challenged with APAP, especially after 6 h and 12 h of treatment, but not in the PMHs without APAP treatment (Fig. 1g). These findings suggested that Egr1 was significantly upregulated in acute AILI models* in vivo* and* in vitro*.

### Egr1 deficiency aggravated acute AILI while Egr1 overexpression ameliorated this

**Fig 2:**
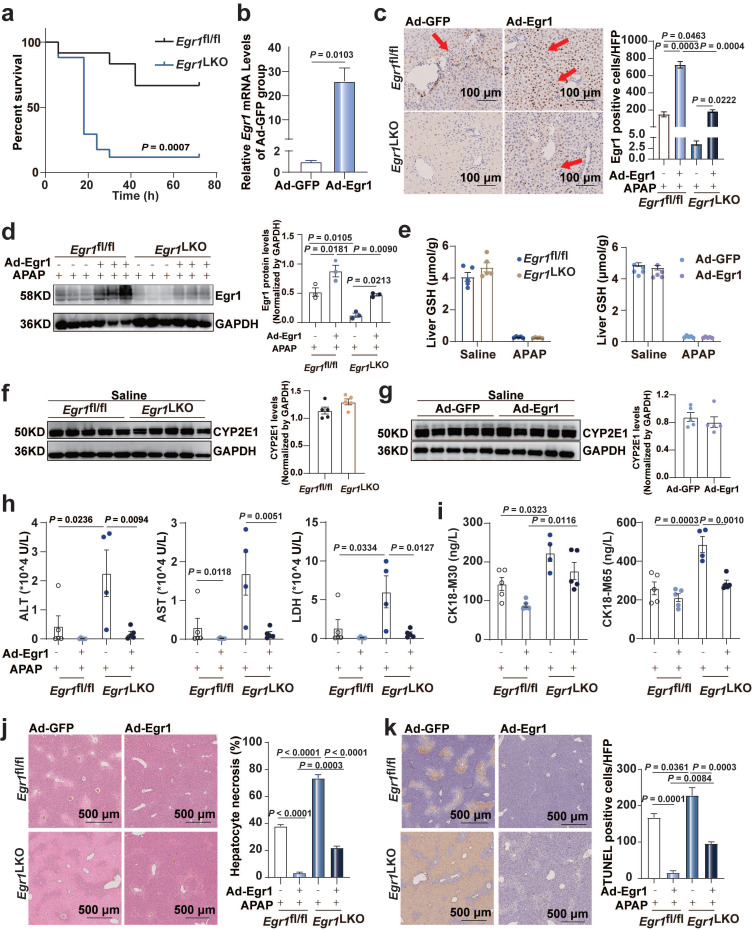
**Egr1 overexpression ameliorated acute AILI.** *Egr1*^fl/fl^ and *Egr1*^LKO^ mice were injected with Ad-Egr1 or Ad-GFP via tail vein prior to 300 mg/kg APAP administration. After 12 h, liver and serum samples were collected. **a.** The survival rate analysis in *Egr1*^fl/fl^ and *Egr1*^LKO^ mice after 750 mg/kg APAP treatment (n = 12 mice/*Egr1*^fl/fl^ group, n = 17 mice/*Egr1*^LKO^ group, Log-rank (Mantel-Cox) test). **b.** Relative *Egr1* mRNA levels in Ad-Egr1 or Ad-GFP pretreated mice (n = 3 mice/group, *t* test). **c.** Immunohistochemical staining images of Egr1 in the liver tissue of Ad-Egr1 or Ad-GFP pretreated AILI *Egr1*^fl/fl^ and *Egr1*^LKO^ mice (scale bar = 100 μm), followed by quantified the numbers of Egr1 positive cells per HPF (n = 3 mice/group, one-way ANOVA). Red arrows represent positive staining. **d.** Western blot analysis of Egr1 levels of total protein in the liver tissues of Ad-Egr1 or Ad-GFP pretreated AILI *Egr1*^fl/fl^ and *Egr1*^LKO^ mice, followed by quantified protein levels (n = 3 mice/group, one-way ANOVA). **e.** Hepatic GSH levels in *Egr1*^fl/fl^ and *Egr1*^LKO^ mice after challenge with saline and APAP for 2 h (n = 5 mice/group, *t* test), and in Ad-Egr1 or Ad-GFP pretreated mice after challenge with saline and APAP for 2 h (n = 5 mice/group, *t* test). **f.** Western blot analysis of CYP2E1 levels in the liver tissues of *Egr1*^fl/fl^ and *Egr1*^LKO^ mice after injection with saline for 2 h, followed by quantified protein levels (n = 5 mice/group, *t* test). **g.** Western blot analysis of CYP2E1 levels in the liver tissues of Ad-Egr1 and Ad-GFP mice after injection with saline for 2 h, followed by quantified protein levels (n = 5 mice/group, *t* test). **h.** Serum ALT, AST, and LDH levels in Ad-Egr1 or Ad-GFP pretreated AILI *Egr1*^fl/fl^ and *Egr1*^LKO^ mice were measured (*n* = 4-5 mice/group, one-way ANOVA). **i.** Serum CK18-M30 and CK18-M65 levels in Ad-Egr1 or Ad-GFP pretreated *Egr1*^fl/fl^ and *Egr1*^LKO^ AILI mice were measured (*n* = 4-5 mice/group, one-way ANOVA). **j.** Liver sample obtained from Ad-Egr1 or Ad-GFP pretreated *Egr1*^fl/fl^ and *Egr1*^LKO^ AILI mice were stained with H&E, followed by quantified the area of hepatocyte necrosis (scale bars = 500 μm, one-way ANOVA). **k.** Liver sample obtained from Ad-Egr1 or Ad-GFP pretreated AILI *Egr1*^fl/fl^ and *Egr1*^LKO^ mice were stained with TUNEL, followed by quantified the numbers of TUNEL positive cells per HFP (scale bars = 500 μm, one-way ANOVA).

To investigate the role of Egr1 in AILI, *Egr1*^LKO^ and *Egr1*^fl/fl^ mice were challenged with 300 mg/kg APAP for 12 h. The mRNA levels of *Egr1* in *Egr1*^LKO^ mice were diminished in the liver tissues but not in the other tissues of these mice, confirming the deletion specificity (Fig. S1a). The mRNA and protein levels of Egr1 were undetectable in the liver tissues of *Egr1*^LKO^ mice (Fig. S1b). The *Egr1*^LKO^ mice exhibited normal growth and development. The survival rates of the *Egr1*^LKO^ mice were significantly decreased in comparison to that of *Egr1*^fl/fl^ mice following the challenge with 750 mg/kg APAP (Fig. 2a). This indicated that the liver-specific deficiency of Egr1 exacerbated AILI. The serum levels of ALT, AST, and LDH were markedly increased in *Egr1*^LKO^ mice compared with those of *Egr1*^fl/fl^ mice following the administration of 300 mg/kg APAP for 12 h (Fig. S1c). These observations were corroborated by the results of H&E staining, which revealed a significant increase in centrilobular hepatic necrosis in the* Egr1*^LKO^ group after APAP treatment for 12 h (Fig. S1d). TUNEL staining used to detect DNA fragmentation, revealed that the treatment of *Egr1*^LKO^ mice with APAP for 12 h resulted in more TUNEL positively-stained hepatocytes, demonstrating that the liver-specific deficiency of Egr1 directly aggravated AILI (Fig. S1e). The effects of liver injury, as observed by investigating the serum (ALT, AST, and LDH) and tissue samples, at 24 h were similar to that at 12h in the* Egr1*^LKO^ mice group (Fig. S1g-h). Two mice in the *Egr1*^Lko^ group died after 19 h and 22 h of APAP challenge. Together, these data implied that Egr1 played a prominent protective role against acute AILI.

To further confirm the protective role of Egr1 in AILI, Ad-Egr1 was injected into *Egr1*^fl/fl^ and *Egr1*^LKO^ mice via the tail vein to overexpress Egr1 before the administration of 300 mg/kg APAP for 12 h. One *Egr1*^LKO^ mouse in the Ad-GFP group died after 10 h of APAP challenge. Egr1 was successfully overexpressed in the hepatocytes by Ad-Egr1 in both *Egr1*^fl/fl^ and *Egr1*^LKO^ mice using RT-qPCR, and immunohistochemical staining, and western blotting (Fig. 2b-d). To rule out the possibility that Egr1 affects APAP metabolism, we detected the hepatic GSH after 2 h of saline and APAP treatment, and the results showed that they decreased markedly after APAP treatment in all groups. The knockout and overexpression of Egr1 did not affect the liver levels of GSH after both the saline and APAP treatment (Fig. 2e). Similarly, CYP2E1 levels were not affected by Egr1 knockout and overexpression after saline (Fig. 2f-g) and APAP challenge (Fig. S1f). The overexpression of Egr1 in the AILI models reduced the APAP-induced elevation in the serum levels of ALT, AST, and LDH in both *Egr1*^fl/fl^ and *Egr1*^LKO^ mice (Fig. 2h). CK18 has been identified as a DILI biomarker. The serum levels of CK18-M30 and M65 were measured by ELISA. As depicted in Fig. 2i, the levels of CK18-M30 and M65 were enhanced by the deletion of Egr1, but reduced by Egr1 overexpression, and the levels of CK18-M65 were higher than those of M30 in all groups. H&E staining revealed that the hepatic centrilobular necrotic area was significantly reduced by the overexpression of Egr1 in the APAP-injured liver tissues (Fig. 2j). Consistently, the numbers of TUNEL-positive hepatocytes were reduced by the overexpression of Egr1 in both *Egr1*^fl/fl^ and *Egr1*^LKO^ mice when challenged with APAP (Fig. 2k). The effects of liver injury, as observed by investigating the serum (ALT, AST, and LDH) and tissue samples, at 24 h were similar to that at 12h in the overexpressed group (Fig.S1i-j). These findings clearly demonstrated that Egr1 was significantly protective against AILI.

### Genome-wide analysis of Egr1 binding sites and analysis of Egr1-induced transcriptomic changes in APAP-injured murine liver

**Fig 3:**
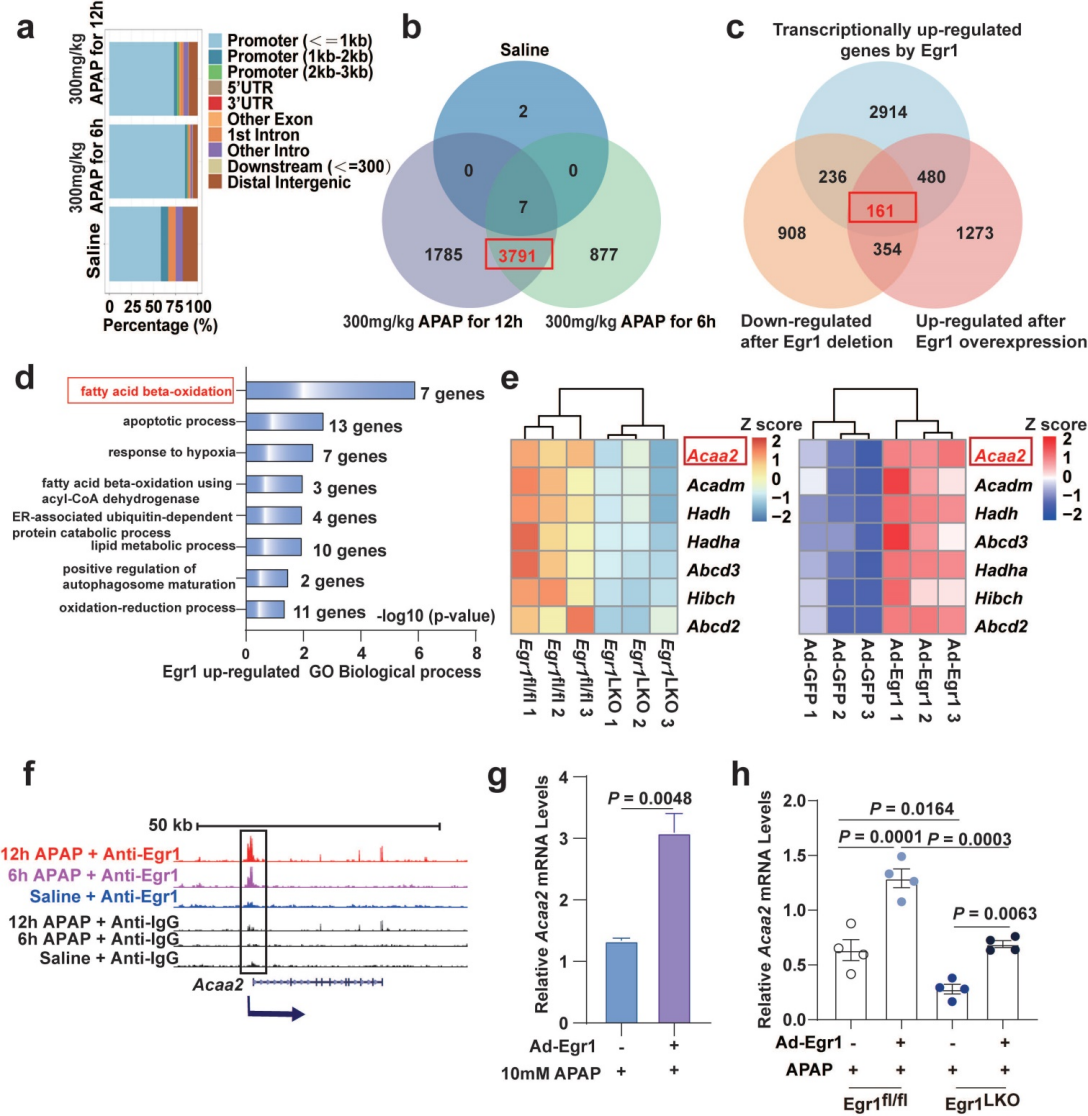
** Genome-wide analysis of Egr1 binding sites and analysis of Egr1 induced transcriptomic changes in APAP-injured mouse liver.** Mice were treated with saline, 300 mg/kg APAP for 6 h and 12 h APAP, and liver samples were collected for ChIP-seq. *Egr1*^fl/fl^ and *Egr1*^LKO^ mice were injected with 300 mg/kg APAP for 12 h, and liver samples were collected for RNA-seq. *Egr1*^LKO^ mice were pretreated with Ad-Egr1 or Ad-GFP prior to challenge with 300 mg/kg APAP, after 12 h the liver samples were collected for RNA-seq. **a.** Bar-plot showed genomic distribution of peaks. **b.** Venn diagram showing the corresponding numbers of *Egr1*-bound genes analyzed from ChIP-seq. **c.** Venn diagram showed the distinct genes that were transcriptionally regulated by Egr1, down-regulated by Egr1 deletion, up-regulated by Egr1 overexpression (fold change ≥ 1.5, P < 0.05, *t* test). **d.** Significantly enriched biological process categories in the GO analysis concerning the 161 transcriptionally upregulated genes. **e.** Heatmaps representing the Z score changes of genes in FAO between *Egr1*^fl/fl^ AILI mice and *Egr1*^LKO^ AILI mice, and Z score changes of genes in FAO between Ad-Egr1 or Ad-GFP pretreated AILI mice (n = 3 mice/group, fold change ≥ 1.5, P < 0.05,* t* test). **f.** Genome-browser screenshots of *Acaa2* occupancy at Egr1 gene loci. **g.** Relative *Acaa2* mRNA levels in AML12 cells of Ad-Egr1 or Ad-GFP group after 10mM APAP challenge for 6 h (*t* test). **h.** Relative *Acaa2* mRNA levels in *Egr1*^fl/fl^ and *Egr1*^LKO^ mice of Ad-Egr1 or Ad-GFP group after 300mg/kg APAP challenge for 12 h (n = 4 mice/group, one-way ANOVA).

To explore the mechanism underlying the protective role of Egr1 against APAP-induced hepatotoxicity, a genome-wide study of Egr1 binding was performed using ChIP-seq, and the changes induced by Egr1 in the global transcriptomic profiles were analyzed by RNA-seq. For ChIP-seq, liver tissues were collected from mice treated with saline, or 300 mg/kg APAP for 6 and 12h. The western blot analysis revealed that the anti-Egr1 antibody specifically pulled down Egr1 (Fig. S2a). The Egr1-ChIP DNA fragments were analyzed using a Genome Analyzer IIx deep sequencer (Illumina). The ChIP-seq raw data yielded 20 million individual sequencing reads. To obtain a genome-wide map of the Egr1 target sites, the obtained tags were mapped onto the mouse genome. We observed that most of the peaks were localized in the gene promoters upstream of the transcription start sites (TSS) (from TSSs to 1 kb upstream of the TSSs) (Fig. 3a). In this study, only 12 peaks and 9 associated genes were identified in the liver tissue of the mouse treated with saline, while 4401 peaks and 3791 associated genes were identified in the liver tissues of mice after both 6 and 12 h of APAP treatment (Fig. 3b and Fig. S2b). These results indicated that 3791 genes were transcriptionally regulated by Egr1 in AILI.

In order to investigate the alterations induced by Egr1 at the transcriptional level, we performed large-scale transcriptomic profiling of APAP-treated *Egr1*^fl/fl^ and *Egr1*^LKO^ mice, or *Egr1*^LKO^ mice pretreated with Ad-Egr1 or Ad-GFP prior to challenge with 300 mg/kg APAP. The heatmaps demonstrated distinct alterations in the pattern of gene expression following *Egr1* deletion or overexpression (Fig. S2c).

In order to identify the target genes upregulated by Egr1, the genes downregulated by the deletion of Egr1, the genes upregulated by the overexpression of Egr1, and the aforementioned 3791 target genes of Egr1 identified by ChIP-seq analysis, were overlapped using a Venn diagram. The results demonstrated that a total of 161 genes in the overlapping area were transcriptionally upregulated by Egr1 in APAP-treated mice (Fig. 3c). We next performed GO enrichment analysis of the 161 genes. As depicted in Fig. 3d, the 161 genes upregulated by Egr1 were mainly enriched in GO biological processes, including “fatty acid beta-oxidation”, “apoptotic process”, and “response to hypoxia”. FAO was identified as the most significant biological process, including seven differential genes (*Acaa2*, *Acadm*, *Hadh*, *Hadha*, *Abcd3*,* Hibch*, and *Abcd2*). Among the seven genes, the expression of *Acaa2* was the most abundant, and *Acaa2* was significantly altered by the deletion of Egr1 (Fig. 3e). The enrichment of these seven genes involved in FAO at Egr1*-*bound loci was significantly increased in the APAP groups compared to that in the saline groups (Fig. 3f and Fig. S2d). The mRNA levels of *Acaa2* were significantly augmented by Egr1 in AML12 cells (Fig. 3g) and murine liver tissues (Fig. 3h).

### The levels of Egr1 affected mitochondrial function in APAP-injured hepatocytes

**Fig 4:**
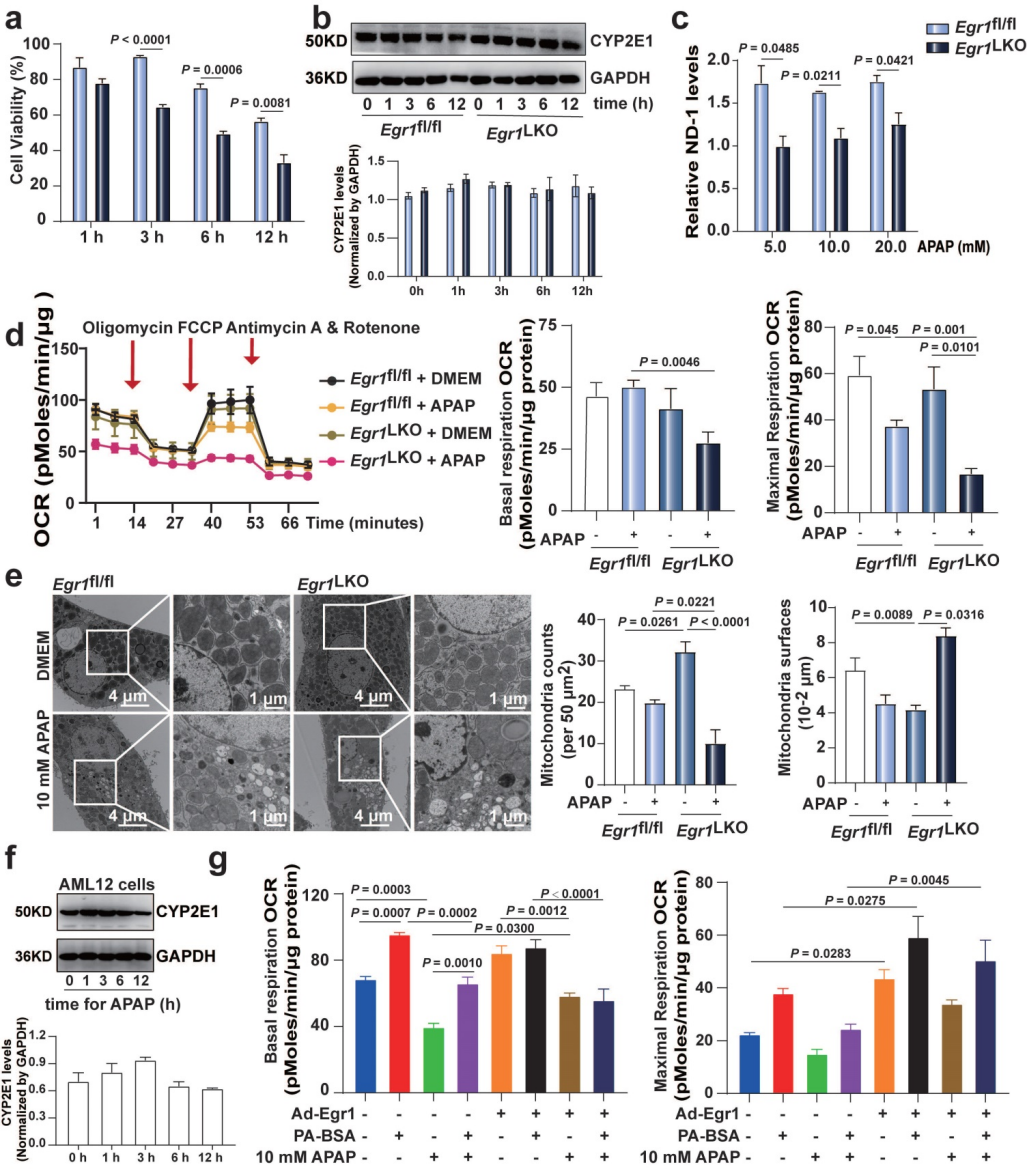
**The levels of Egr1 affected mitochondrial function in APAP-injured hepatocytes. a.** PMHs were isolated from *Egr1*^fl/fl^ and *Egr1*^LKO^ mice and then treated with 20 mM APAP for different times. Cell viability was measured by CellTiter-Glo® luminescent cell viability assay (*t* test). **b.** Western blot analysis of CYP2E1 levels in PMHs from *Egr1*^fl/fl^ and *Egr1*^LKO^ mice treated with 20 mM APAP for different times, followed by quantified protein levels (*t* test). **c.** The ND-1 levels of PMHs treated with different doses of APAP for 12 h in *Egr1*^fl/fl^ and *Egr1*^LKO^ groups (*t* test).** d.** Cell mito stress OCRs cause by *Egr1* deficiency in PMHs were measure by Seahorse XF96 analyzer. Basal and maximal respiration were calculated according to instruction. Red arrows indicated the times when oligomycin, FCCP, and antimycin/rotenone were added to PMHs (*n* = 4/group, one-way ANOVA). **e.** Representative TEM images of PMHs in *Egr-1*^LKO^ and *Egr-1*^fl/fl^ groups after DMEM (for control) and APAP treatment. Corresponding relative mitochondrial counts and surfaces were analyzed (one-way ANOVA). **f.** Western blot analysis of CYP2E1 levels in AML12 cells treated with 20 mM APAP at different times, followed by quantified protein levels (one-way ANOVA). **g.** AML12 cells were treated with Ad-Egr1 or Ad-CON for 48 h and then challenged with 10 mM APAP for 6 h, followed by PA-BSA or BSA treated for 1 h. Palmitate oxidation stress OCRs were measured using Seahorse XF96 analyzer. Basal and maximal respiration were calculated according to instruction (n = 4/group, one-way ANOVA). BSA was used as a control for PA-BSA.

As FAO primarily occurs inside the mitochondria, and as mitochondria play a central role in APAP-induced cell death, we investigated the role of Egr1 in APAP-induced mitochondrial dysfunction. As depicted in Fig. 4a, the viability of PMHs was significantly decreased after 3, 6, and 12 h of treatment with 20 mM APAP in the* Egr1*^LKO^ group, compared to that of the* Egr1*^fl/fl^ group. The levels of CYP2E1 were not affected in PMHs by Egr1 levels (Fig. 4b). The levels of ND-1, the mitochondrial DNA, were decreased in the *Egr1*^LKO^ group following APAP challenge (Fig. 4c). Cell mito stress oxygen consumption rates (OCRs) were measured by Seahorse XF analysis. The basal and maximal mitochondrial OCRs were markedly reduced after 10 mM APAP challenge in the* Egr1*^LKO^ group, compared to those of the* Egr1*^fl/fl^ group (Fig. 4d). Transmission electron microscopy revealed that the numbers of mitochondria were decreased, while the mitochondrial area was increased in the* Egr1*^LKO^ group (Fig. 4e). In order to further explore the effect of Egr1 on FAO capacity, we examined the PA-based OCR. The western blotting analysis revealed that AML12 cells could express CYP2E1 to metabolize APAP (Fig. 4f). The PA-based OCR showed that overexpression of Egr1 increased the basal and maximal mitochondrial OCRs, and enhanced the capacity of FAO in AML12 cells (Fig. 4g and Fig. S3). Taken together, these findings suggest that Egr1 might alleviate the mitochondrial dysfunction caused by APAP and enhance the capacity of FAO.

### Egr1 deficiency increased lipid droplet accumulation and FFAs levels in AILI

**Fig 5:**
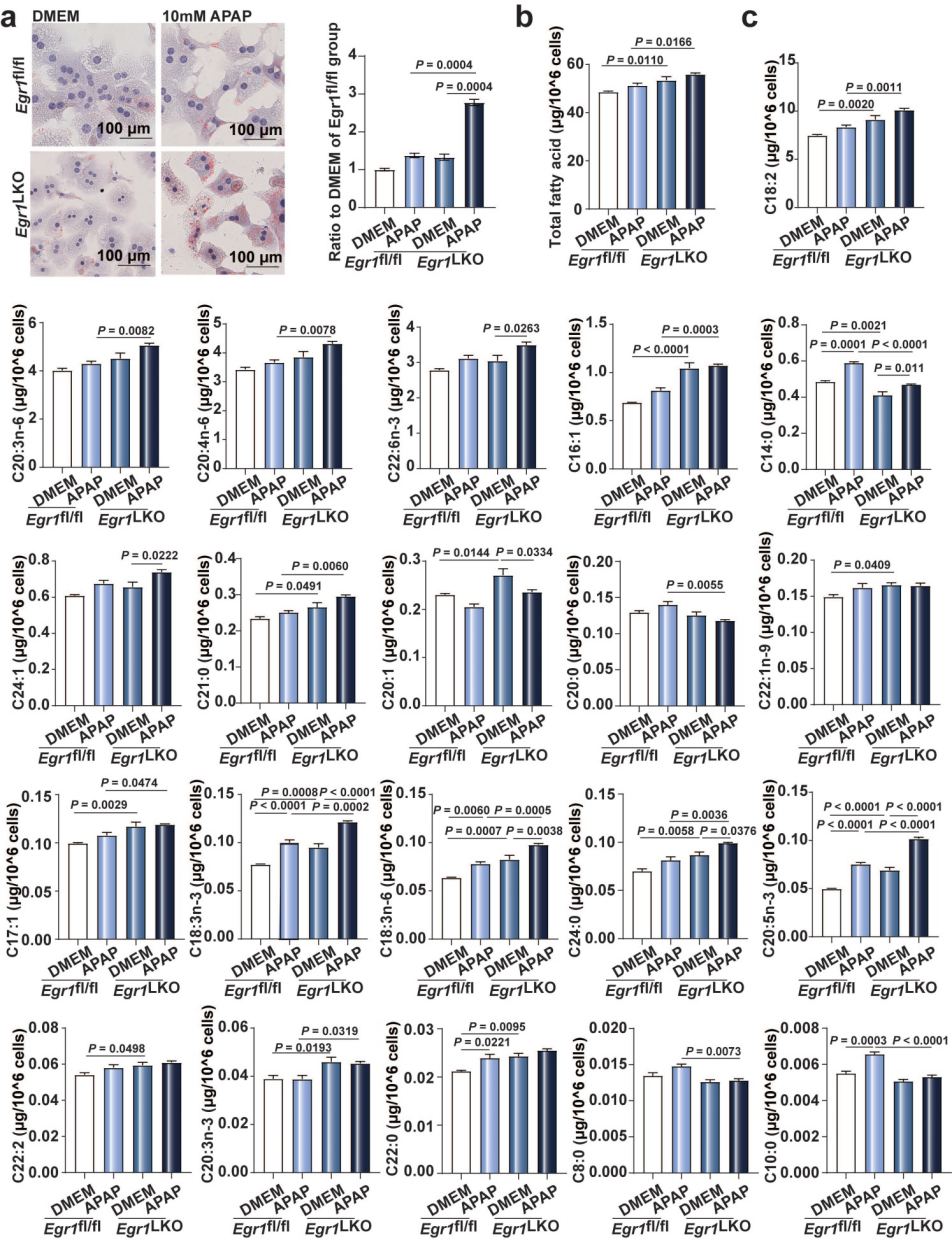
** Egr1 deficiency increased lipid droplets accumulation and FFAs levels in AILI. a.** Oil Red O staining of PMHs in *Egr1*^fl/fl^ and *Egr1*^LKO^ groups after DMEM or 10 mM APAP treatment and the quantification of the ratio relative to the DMEM of *Egr1*^fl/fl^ group (one-way ANOVA). **b-c.** The levels of total fatty acids (**b**) and each fatty acid (**c**) of PMHs in *Egr1*^fl/fl^ and *Egr1*^LKO^ groups after 10 mM APAP treatment for 12 h determined by GC-MS-based FFA analysis (one-way ANOVA).

Microvesicular steatosis, the accumulation of numerous small lipid droplets in the hepatocytic cytoplasm, is also a characteristic feature of AILI. As depicted in Fig. 5a, Oil Red O staining revealed that the accumulation of small lipid droplets was increased in the *Egr1*-deficient hepatocytes, compared to that of the control cells following exposure to 10 mM APAP. We additionally measured the levels of FFAs in the primary hepatocytes from *Egr1*^fl/fl^ and *Egr1*^LKO^ mice using GC-MS. As depicted in Fig. 5b, the levels of total FFAs increased in the *Egr1*^LKO^ group with APAP challenge, in comparison to those of the* Egr1*^fl/fl^ group. Meanwhile, the levels of C14:0, C21:0, C18:2, C18:3n-3, C20:4n-6, C20:3n-6, C20:5n-3, and C22:6n-3 were dramatically enhanced following the deletion of *Egr1* in the hepatocytes (Fig. 5c). Together, these data revealed that the deficiency of *Egr1* increased the accumulation of small lipid droplets and the levels of FFAs in AILI.

### *Egr1* protected mice and hepatocytes against AILI by transcriptionally upregulating *Acaa2*

**Fig 6:**
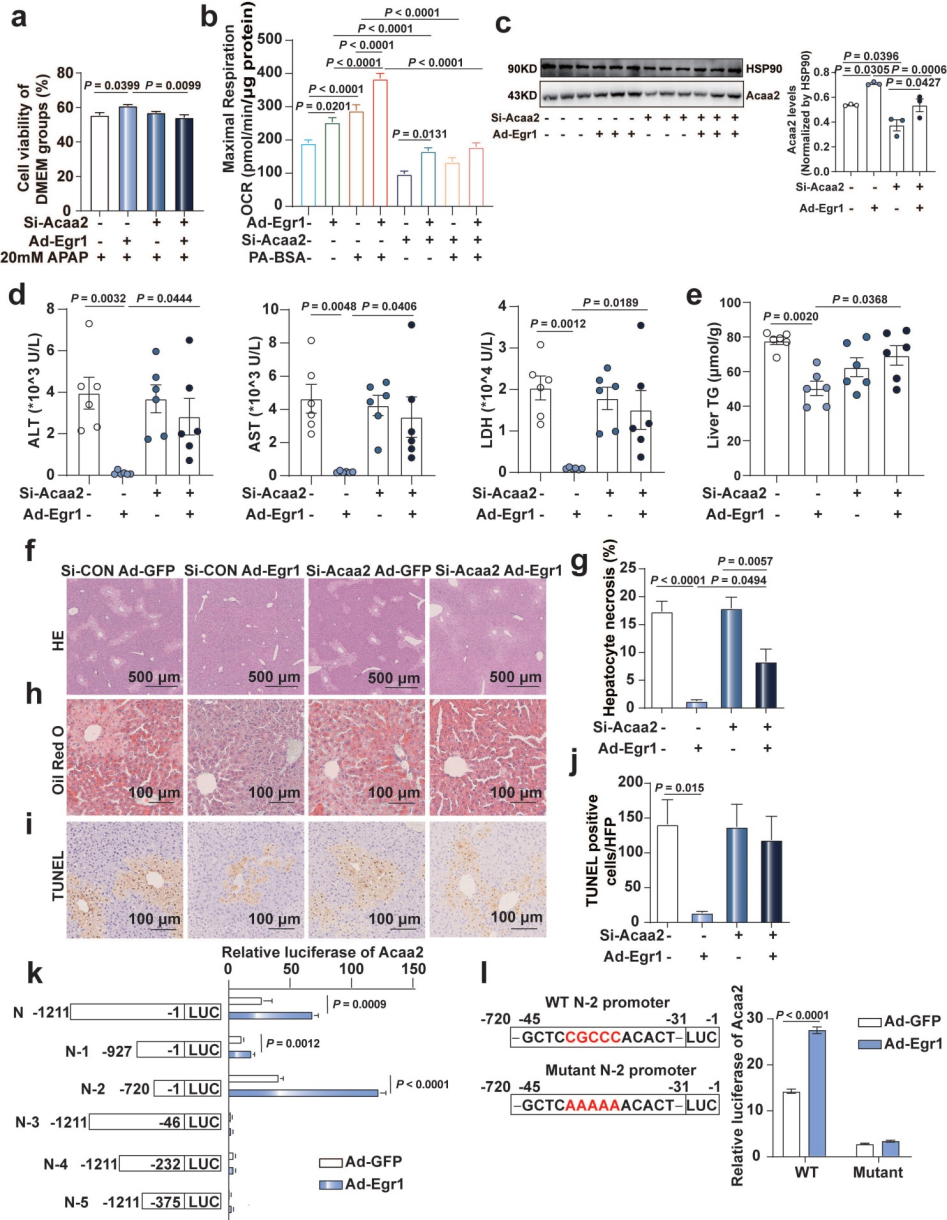
**Egr1 protect mice and hepatocytes against AILI by transcriptionally up-regulating Acaa2. a.** AML12 cells were knocked down of Acaa2 for 24 h, then overexpressed Egr1 for 24 h, finally challenged with 20 mM APAP treatment for 24 h. Cell viability was measured by CCK8 assay (one-way ANOVA). Cell viability of DMEM groups was used as normalization control groups. **b.** Acaa2 was knocked down in Hepa1-6 cells at 24 h, then overexpressed Egr1 for 48 h and followed by 10 mM APAP treatment for 3 h, finally PA-BSA or BSA treated for 1 h. Palmitate oxidation stress OCRs were measured using Seahorse XF96 analyzer. Maximal respiration was calculated according to instruction (n = 5-6/group, one-way ANOVA). BSA was used as a control for PA-BSA. **c**-**j.** Mice were knocked down of Acaa2 at 24 h, then overexpressed Egr1 for 48 h via tail vein prior to 300 mg/kg APAP administration. After 6 h, liver and serum samples were collected. **(c)** Western blot analysis of Acaa2 levels in liver tissues of all groups, followed by quantified protein levels (n=3 mice/group, one-way ANOVA). Serum ALT, AST, LDH (**d**) levels, liver TG (**e**), HE (**f** and **g**), Oil Red O staining (**h**) and TUNEL staining (**i** and **j**) in all mice groups (n = 6 mice/group, one-way ANOVA). **k.** Luciferase activity assay of *Acaa2* N-terminal promoter and truncated N-terminal promoters (N-1-5) in AML12 cells after Ad-Egr1 or Ad-CON treatment (*t* test). **l.** Luciferase activity assay of WT and mutant N-2 promoters in AML12 cells after Ad-Egr1 or Ad-CON treatment (*t* test).

As depicted in Figure 3, *Acaa2* was the most significantly altered FAO gene regulated by Egr1. We therefore investigated the key role of *Acaa2* in the protective function of Egr1 in AILI. The results of CCK8 assays revealed that the increase in cell viability caused by Egr1 was diminished by the knockdown of Acaa2 using si-Acaa2 in AML12 cells (Fig. 6a). As depicted in Fig. S4a, Hepa1-6 cells could express CYP2E1 to metabolize APAP. The increase in PA-based basal and maximal mitochondrial OCRs following the overexpression of Egr1 was also diminished by si-Acaa2 in Hepa1-6 cells (Fig. 6b and Fig. S4b).

C57BL/6J mice were knocked down of Acaa2, then overexpressed Egr1 prior to 300 mg/kg APAP administration for 6 h. Si-Acaa2 successfully reduced the expression of Acaa2 in the liver tissues of mice (Fig. 6c). The reduction levels of ALT, AST, and LDH in the serum and TG levels in the liver tissues caused by the adenovirus-mediated overexpression of Egr1, were diminished by si-Acaa2, compared to that of si-CON mice, following the administration of APAP (Fig. 6d-e). H&E, Oil Red O, and TUNEL staining of murine liver tissues revealed that hepatocytic necrosis, the accumulation of small lipid droplets, and DNA fragmentation were not attenuated by the overexpression of Egr1 in si-Acaa2 treated mice (Fig. 6f-j). In order to further elucidate the binding sites of Egr1 in the promoter region of *Acaa2*, fragments of the *Acaa2* promoter (N and N-1~5) were cloned into the pGL4 luciferase enhancer vector. Following the overexpression of Egr1, the Acaa2 reporters were transfected, and the luciferase activity was monitored in AML12 cells. As depicted in Fig. 6k, the overexpression of Egr1 enhanced the transcriptional activity of N, N-1, and N-2 luciferase reporters containing the Egr1 binding site, which revealed that the Egr1 motif was located N-2 region (-1 to -720 bp) of the translational start site of *Acaa2*. Moreover, mutations at the N-2 region abolished the increased transcriptional activity, confirming that the -31 to -45 bp region is responsible for Egr1 binding (Fig. 6l). These results indicated that Egr1 protected hepatocytes against AILI via the transcriptional upregulation of *Acaa2*.

### Levels of EGR1 were increased in the liver and serum samples of patients with AILI/DILI

We detected protein expression in the liver tissues and serum samples from two separate DILI cohorts. Only three AILI patient liver biopsy samples were available for analysis. We observed that EGR1 H-scores of liver were markedly higher in these three AILI patients (4.083 ± 3.660) compared with those in healthy controls (0.3125 ± 0.470) (*P* = 0.0003). This finding was consistent with the results *in vivo* and *in vitro*.

**Fig 7:**
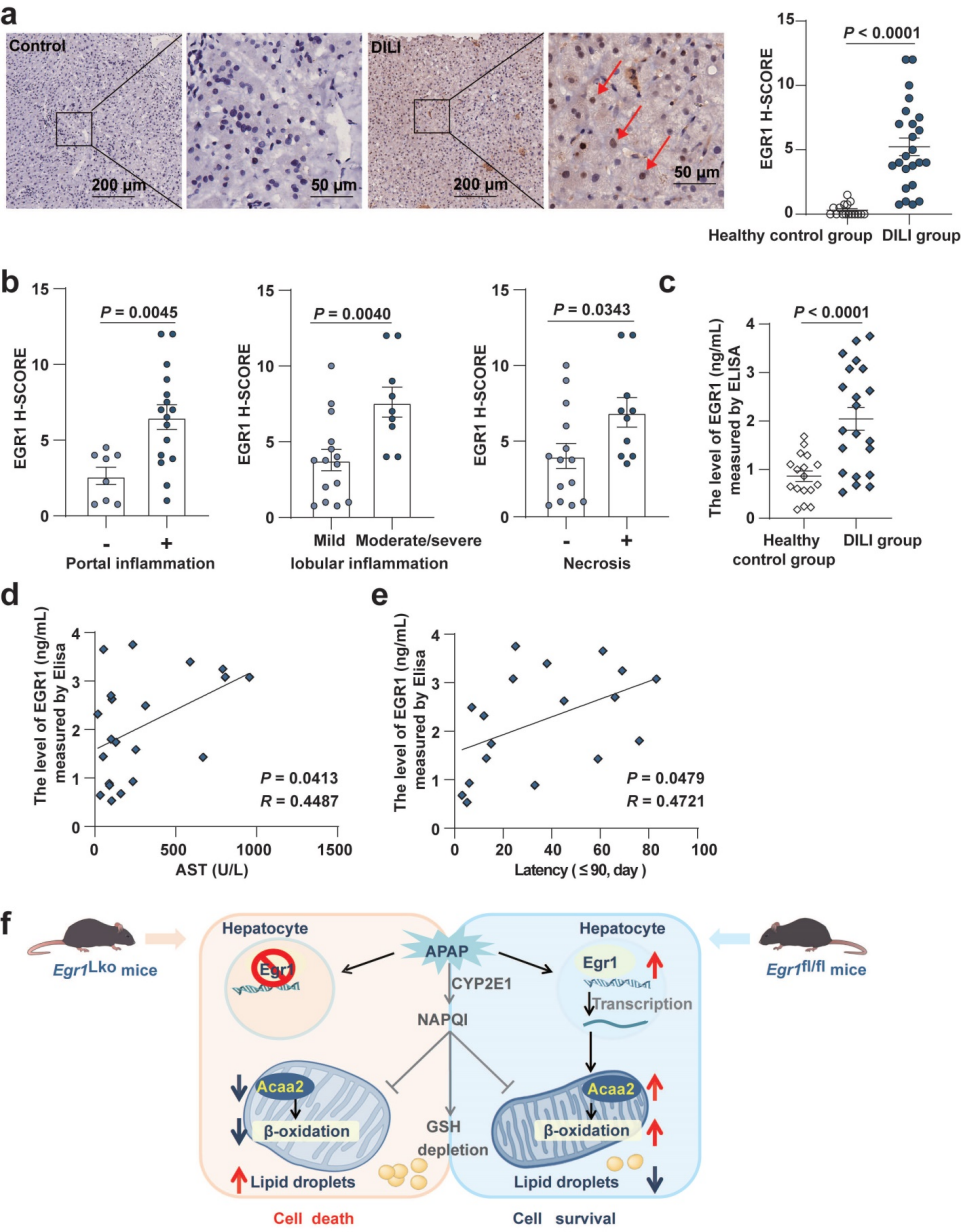
**Levels of EGR1 were increased in the liver and serum samples of patients with DILI. a.** Representative EGR1 staining patterns in liver samples from DILI patients and healthy controls (scale bars = 200 μm or 50 μm). EGR1 H-scores in liver samples obtained from 24 patients with DILI and 16 healthy controls (*t* test). Red arrows indicated positive staining. **b.** Distribution of liver histopathologic features and corresponding EGR1 H-scores in patients with DILI (*t* test). **c.** The levels of EGR1 in serum samples from 21 patients with DILI and 17 healthy controls were measured by ELISA (*t* test). **d.** Pearson correlation was performed between serum AST levels in 21 DILI patients and EGR1 serum levels. **e.** Pearson correlation was performed between latency less than 90 days in 18 DILI patients and EGR1 serum levels. **f.** Graphical abstract.

Interestingly, compared with the healthy control group, we found EGR1 levels were upregulated not only in AILI patients but also in DILI patients induced by different implicated agents. EGR1 was found to be strongly expressed in the nuclei of hepatocytes in patients with AILI/DILI but not in tissues from the healthy control group (Fig. 7a). Moreover, EGR1 levels in liver were significantly higher in AILI/DILI patients with specific histopathologic features, such as portal inflammation (*P* = 0.0045), moderate-to-severe lobular inflammation (*P* = 0.0040), or necrosis (*P* = 0.0343) (Fig. 7b and Fig. S5a). However, there was no difference in EGR1 staining between AILI/DILI patients with and without interface hepatitis (*P* = 0.0789) and cholestasis (*P* = 0.9882) (Fig. S5b-c). Taken together, we demonstrated that levels of EGR1 were obviously upregulated in liver samples of patients with AILI/DILI.

Moreover, the results of ELISA revealed that the levels of EGR1 in serum were higher in the 21 patients with DILI than in the 17 healthy controls (*P* < 0.0001) (Fig. 7c). Pearson correlation analysis showed that the serum levels of EGR1 were positively correlated with the levels of AST (*R* = 0.4487, *P* = 0.0413; Fig. 7d) and the latency of DILI when the latency was within 90 days (*R* = 0.4721, *P* = 0.0479; Fig. 7e).

## Discussion

The present study demonstrated that the EGR1 levels were elevated in acute AILI models both *in vivo* and *in vitro*. Upregulated Egr1 levels were also seen in the livers and serum samples of patients with AILI/DILI. The liver-specific deletion of Egr1 aggravated, but the overexpression alleviated AILI. Egr1 deficiency inhibited mitochondrial respiration and fatty acids β-oxidation, whereas its overexpression had the opposite effects. Using RNA-seq, ChIP-seq analysis, and functional experiments, we found that Acaa2 mediated the protective role of Egr1 in AILI.

Few studies reported the expression and effect of Egr1 on DILI. Data mining from the TG-GATEs (Toxicogenomics Project-Genomics Assisted Toxicity Evaluation system) database revealed that *Egr1* was one of the four genes to be significantly upregulated (*Egr1*, *Atf3*, *Gdf15*, and *Fgf21*) at the early stage of DILI 39. Similarly, we observed that the mRNA and protein levels of Egr1 were upregulated in AILI both *in vivo* and *in vitro*. The mRNA levels of *Egr1* were significantly increased even at the early stage after APAP challenge, indicating its rapid response to the APAP challenge. Interestingly, we observed that the positive staining of nuclear Egr1 was enhanced in the hepatocytes located at the boundary of the necrotic area in AILI, but not in the necrotic hepatocytes. These results suggested that the increased expression of Egr1 might be an adaptive protective response to APAP-induced injury in hepatocytes. Currently, two inconsistent studies have reported the effect of Egr1 on APAP-induced hepatotoxicity. Pang et al reported that when L02 cells were transiently transfected with Egr1 siRNA, the decreased cell viability induced by APAP could be reversed 26. However, Bai et al reported that compared with wild-type mice, Egr1 knock-out mice suffered more serious APAP-induced liver fibrosis due to the higher levels of TGF-β, α-smooth muscle actin, and collagens 27. In our study, we utilized the liver-specific Egr1 knockout mice, primary hepatocytes, AML12 cells, Hepa1-6 cells, and adenovirus Egr1 to explore the role of Egr1 in AILI. All of our models consistently showed that Egr1 could protect against APAP-induced hepatotoxicity. Considering that different cell line was used in the studies of Pang et al, the different cell lines may result in different effects of Egr1 in AILI. Although we did not identify the APAP-adduct in our study, published literature indicates that the APAP-adduct formation is determined by CYP2E1 and GSH levels 40, 41. In our study, neither Egr1 knockout nor overexpression affected the levels of CYP2E1 and GSH, suggesting that Egr1 might not affect the APAP-adduct. These findings suggest that Egr1 plays an important role in adaptive protection in acute AILI.

In our study, the* Egr1*^LKO^ mice were indeed severely injured, several mice in *Egr1*^LKO^ died before euthanized. These suggesting the important protective role of Egr1 in acute AILI. Cell death in the AILI occurs mainly by necrosis and apoptosis^42^. The CK18-M65 is present both on the full-length and cleaved form of K18 (necrosis and apoptosis), whereas the CK18-M30 is formed on the K18 fragments during apoptotic cleavage. The elevated levels of CK18-M30 and M65 could be observed in our study indicating both apoptosis and necrosis ongoing, these were consistent with the previous studies 15, 43.

FAO is a multi-step metabolic process in which fatty acids are shortened to acetyl-coenzyme A which could next be oxidized via the tricarboxylic acid cycle 44. The impairment of mitochondrial respiration and FAO are pivotal mechanisms underlying DILI, including AILI 45-49. Previous studies have reported that the activation of peroxisome proliferator-activated receptor α (PPARα), which controls the expression of genes encoding mitochondrial FAO enzymes, can protect against AILI in murine models 50, 51. Microvascular fatty liver, a distinctive form of DILI, results from insufficient FFA oxidation and is characterized by the accumulation of small lipid vesicles 44. However, the underlying relationship between the expression of Egr1 and mitochondrial FAO in disease conditions has been scarcely investigated. In our study, the results of ChIP-seq and RNA-seq revealed integrative genome-wide changes resulting from alterations in the expression of Egr1 in AILI. These data demonstrated that the most significant biological process was FAO which is very pivotal in AILI, suggesting that the role of Egr1 is very important and worth exploring. We then further demonstrated that the deficiency of Egr1 suppressed basal and maximal mitochondrial OCRs. Overexpression of Egr1 enhanced FAO capacity in hepatocytes. Analyses of the data obtained from Oil Red O staining and GC-MC further revealed that the deficiency of Egr1 significantly suppressed FAO and promoted lipid accumulation. These results strongly implied that Egr1 exhibited protective effects against AILI by enhancing mitochondrial FAO.

The *Acaa2* gene encodes an enzyme of the thiolase family, which catalyzes the last step of the respective β-oxidation pathway 52. Li et al. reported that the expression levels of Acaa2 were significantly higher in the fatty liver of obese patients than those of normal controls 53. Takahir et al. reported that the knockdown of Acaa2 increased the proliferation of human hepatocellular carcinomas cells 54. However, the function of Acaa2 in AILI has not been investigated to date, and the relationship between expression of Egr1 and Acaa2 remains to be elucidated. In our study, the knockdown of Acaa2, which is the most distinctly upregulated FAO gene by Egr1, diminished the protective effect of Egr1 on AILI both *in vivo* and *in vitro*. The overexpression of Egr1 did not improve mitochondrial FAO following the Acaa2 knockdown. ChIP-seq and luciferase reporter assay revealed Egr1 transcriptionally upregulated the expression of Acaa2. These findings suggested that Egr1 could transcriptionally upregulate the levels of Acaa2, resulting in the enhancing the mitochondrial FAO to exert its protective effect in AILI. In our study, knockdown Acaa2 alone *in vivo* and *in vitro* did not further increase liver injury compared to APAP alone. The reason is not clear. The remaining high level of Acaa2 and activating other adaptive protection mechanisms after Acaa2 knockdown might be the possible reasons. Meanwhile, the more severe liver injury might be observed when we extended the experiment beyond 6 hours.

Our data revealed, for the first time, that the levels of EGR1 were increased in the hepatocytic nuclei of liver biopsies from three AILI patients. This was consistent with the findings in AILI mouse models, indicating that Egr1 may play an important role during AILI events. In this study, we also found that the levels of EGR1 were markedly increased both in the liver and serum of DILI patients induced by other implicated drugs. The reason why EGR1 was increased in both AILI patients and idiosyncratic DILI patients is unclear. We speculated that the adaptive protective effect of EGR1 may be a common mechanism in the pathogenesis of AILI and DILI. Further research is needed.

There are some limitations to this study. First, the dynamic of Egr1 expression in this study is insufficient. Second, we did not conduct the experiment of Acaa2 overexpression in the *Egr1*^LKO^ mice, which needs further explored. Third, the sample size of AILI patients in this study is small, which needs further validation from a large number of AILI cases. Fourth, liver and serum samples were from different patients in our DILI cohorts. Further studies are needed to investigate the EGR1 levels in the liver and serum from the same patients.

In conclusion, our current study demonstrated that Egr1 played a protective role against acute AILI. The protective effects of Egr1 on AILI could be attributed to its transcriptional upregulation of Acaa2, which improved mitochondrial FAO. The data suggested that the Egr1 was a potential biomarker and therapeutic target for AILI.

## Supplementary Material

Supplementary figures and tables.

## Abbreviations

APAP: acetaminophen
AILI: APAP-induced liver injury
DILI: drug-induced liver injury
Egr1: Early growth response 1
*Egr1* ^LKO^: liver specific Egr1 knockout
Ad-Egr1: adenoviruses Egr1
FAO: fatty acid β-oxidation
Acaa2: acetyl-Coenzyme A acyltransferase 2
NAPQI: N-acetyl-p-benzoquinone imine
CYP: cytochrome P450
GSH: glutathione
*Egr1* ^fl/fl^: homozygous floxed *Egr1*
siRNA: small interfering RNA
ALT: alanine aminotransferase
AST: aspartate aminotransferase
LDH: lactate dehydrogenase
TUNEL: terminal dUTP nick‑end labeling
PMH: primary mouse hepatocyte
TG: triglyceride
H-score: histological score
RT-qPCR: real-time polymerase chain reaction
BSA: bovine serum albumin
PA: palmitate
ChIP-Seq: chromatin immunoprecipitation-sequencing
RNA-Seq: RNA-sequencing
GO: gene ontology
TSS: transcription start sites
OCR: oxygen consumption rate
FFA: free fatty acid
