# Supplementary material for: Egr1 confers protection against acetaminophen‑induced hepatotoxicity via transcriptional upregulating of Acaa2
Source: Int J Biol Sci. 2022 May 29;18(9):3800–17. doi: 10.7150/ijbs.71781 (PMC9254463; doi:10.7150/ijbs.71781)
Supplement: Supplementary file 1 — Supplementary figures and tables. [file ijbsv18p3800s1.pdf]

Supplementary material

Egr1 confers protection against drug-induced hepatotoxicity via transcriptional upregulating of Acaa2

Xiaohong Lei, Qingling Xu, Chunmin Li, Baolin Niu, Yanan Ming, Jing Li, Yingyue Tang, Xiaoyun Li, Jieting Tang, Jing Wu, Yi Ju, Lvfeng Yao, Bin Wang, Qi Miao, Wei Zhong, Yang zhi, Lirong Xu, Chaojun Li, Xiaobo Li, Yimin Mao

Correspondence

Address correspondence to:

Yimin Mao, M.D

Division of Gastroenterology and Hepatology; Shanghai Institute of Digestive Disease; NHC Key Laboratory of Digestive Diseases; Renji Hospital, School of Medicine, Shanghai Jiao Tong University, Shanghai 200001, China. Email: maoym11968@163.com.

Xiaobo Li, Ph.D

Department of Physiology and Pathophysiology, School of Basic Medical Sciences, Fudan University, 130 Dong'an Rd, Shanghai, 200032, China. Email: xbli@fudan.edu.cn.

Chaojun Li, Ph.D

State Key Laboratory of Reproductive Medicine, Centre for Global Health, School of Public Health, Nanjing Medical University, Nanjing, 211100, China Email: lichaojun@njmu.edu.cn.

Supplementary Tables and Figures

Table S1

Characteristics and Clinical data of the DILI patients for IHC

	DILI
**Sex**	
Male	10
Female	14
**Age**	53.13±10.13
**Implicated drugs in causing DILI**	
TCM/HDS	17
NSAID	6
Ibuprofen	1
Diclofenac	1
APAP	3
Details unknown	1
Cold medication (details unknown)	1
**Latency (day)**	36.67±27.93
**Liver biochemistry at onset of DILI**	
ALT (U/L)	1445.73±2686.27
AST (U/L)	637.22±598.63
ALP (U/L)	148.63±80.98
TBIL (μmol/L)	115.5±107.26

HDS, herbal and dietary supplements; TCM, traditional Chinese medicines; Age, latency and liver biochemistry are shown as mean ± SEM

Table S2

Characteristics and Clinical data of the DILI patients for ELISA

	DILI
**Sex**	
Male	9
Female	12
**Age**	54±16.02
**Implicated drugs in causing DILI**	
TCM/HDS	13
Antituberculosis drugs	2
Antitumor drug	3
Fluose fine hydrochloride	1
Cefaclor	1
Cyclosporine	1
**Latency (day)**	44.29±33.43
**Liver biochemistry at onset of DILI**	
ALT (U/L)	728.77±525.38

AST (U/L)	394.62±321.40
ALP (U/L)	198.38±166.53
TBIL (μmol/L)	20.63±13.10

HDS, herbal and dietary supplements; TCM, traditional Chinese medicines; Age, latency and liver biochemistry are shown as mean ± SEM

Table S3

Primer sets used for qPCR

**Species**	**Gene**	**Forward (Sequence 5'-3')**	**Reverse (Sequence 5'-3')**
mouse	*36b4*	GGGCATCACCACGAAAATCTC	CTGCCGTTGTCAAACACCT
mouse	*Gapdh*	TGAAGGTCGGTGTGAACGG	CGTGAGTGGAGTCATACTGGAA
mouse	*Egr1*	GTCCTTTTCTGACATCGCTCTGA	CGAGTCGTTTGGCTGGGATA
mouse	*Acaa2*	AAGAAAGGCAAACAGACCA	AGAACTGAGGGGCAAAAGC
mouse	*ND-1*	CCGGCCCATTCGCGTTATTCTTTA	AAGCGTGGATAGGATGCTCGGATT
mouse	*Egr1 exon2*	CCAACAGCCCTTTCACTTA	TTATGCCAACTTGATGGTCTA

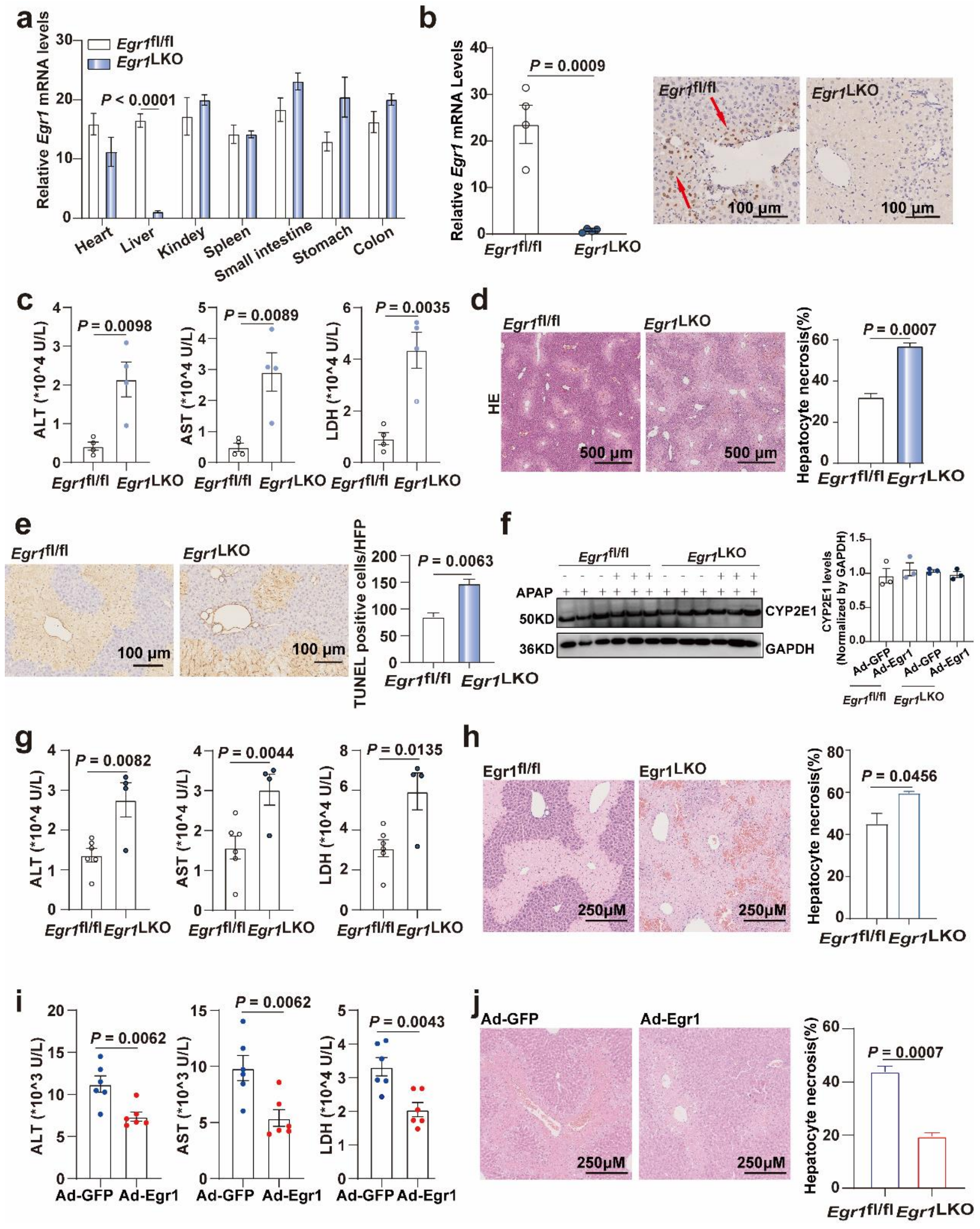

Fig. S1

a Relative *Egr1* mRNA levels in different tissues of *Egr1*^{LKO} mice (n=3 mice/group, t test).

b Relative *Egr1* mRNA levels in liver tissue of *Egr1*$^{fl/fl}$ and *Egr1*^{LKO} AILI mice (n=4 mice/group, t test). Immunohistochemical staining images of Egr1 in the liver tissue of

AILI *Egr1*$^{\mathrm{fl/fl}}$ and *Egr1*^{LKO} mice (scale bar = 100 μm). Red arrows represent positive staining.

c–e *Egr1*$^{\mathrm{fl/fl}}$ and *Egr1*^{LKO} mice were treated with 300 mg/kg APAP. After 12 h, liver and serum samples were collected.

c Serum ALT, AST, and LDH levels in Egr1$^{\mathrm{fl/fl}}$ and Egr1^{LKO} mice after challenge with APAP for 12 h (n = 4 mice/group, *t* test).

d Liver sample obtained from AILI *Egr1*$^{\mathrm{fl/fl}}$ and *Egr1*^{LKO} mice were stained with H&E, followed by quantified the area of hepatocyte necrosis (scale bars = 500 μm, *t* test).

e Liver sample obtained from *Egr1*$^{\mathrm{fl/fl}}$ and *Egr1*^{LKO} AILI mice were stained with TUNEL, followed by quantified the numbers of TUNEL positive cells (scale bars = 500 μm, *t* test).

f Western blot analysis of CYP2E1 levels in the liver tissues of Ad-Egr1 or Ad-GFP pretreated AILI *Egr1*$^{\mathrm{fl/fl}}$ and *Egr1*^{LKO} mice, followed by quantified the protein levels (n=3 mice/group, one-way ANOVA).

g–h *Egr1*$^{\mathrm{fl/fl}}$ and *Egr1*^{LKO} mice were treated with 300 mg/kg APAP. After 24 h, liver and serum samples were collected.

g Serum ALT, AST, and LDH levels in *Egr1*$^{\mathrm{fl/fl}}$ and *Egr1*^{LKO} mice after challenge with APAP for 24 h (n = 6 mice/*Egr1*$^{\mathrm{fl/fl}}$ group, n=4 mice/*Egr1*^{LKO} group, *t* test).

h Liver sample obtained from AILI *Egr1*$^{\mathrm{fl/fl}}$ and *Egr1*^{LKO} mice were stained with H&E, followed by quantified the area of hepatocyte necrosis (scale bars = 250 μm, *t* test).

i–j C57BL/6J mice were injected with adenovirus encoding *Egr1* (Ad-Egr1) or control (Ad-GFP) via tail vein prior to 300 mg/kg APAP administration. After 24 h, liver and

serum samples were collected.

i Serum ALT, AST, and LDH levels of Ad-Egr1 and Ad-GFP mice after challenge with APAP for 24 h (n = 6 mice/ group, *t* test).

j Liver sample obtained from Ad-Egr1 and Ad-GFP mice were stained with H&E, followed by quantified the area of hepatocyte necrosis (scale bars = 250 μm, *t* test).

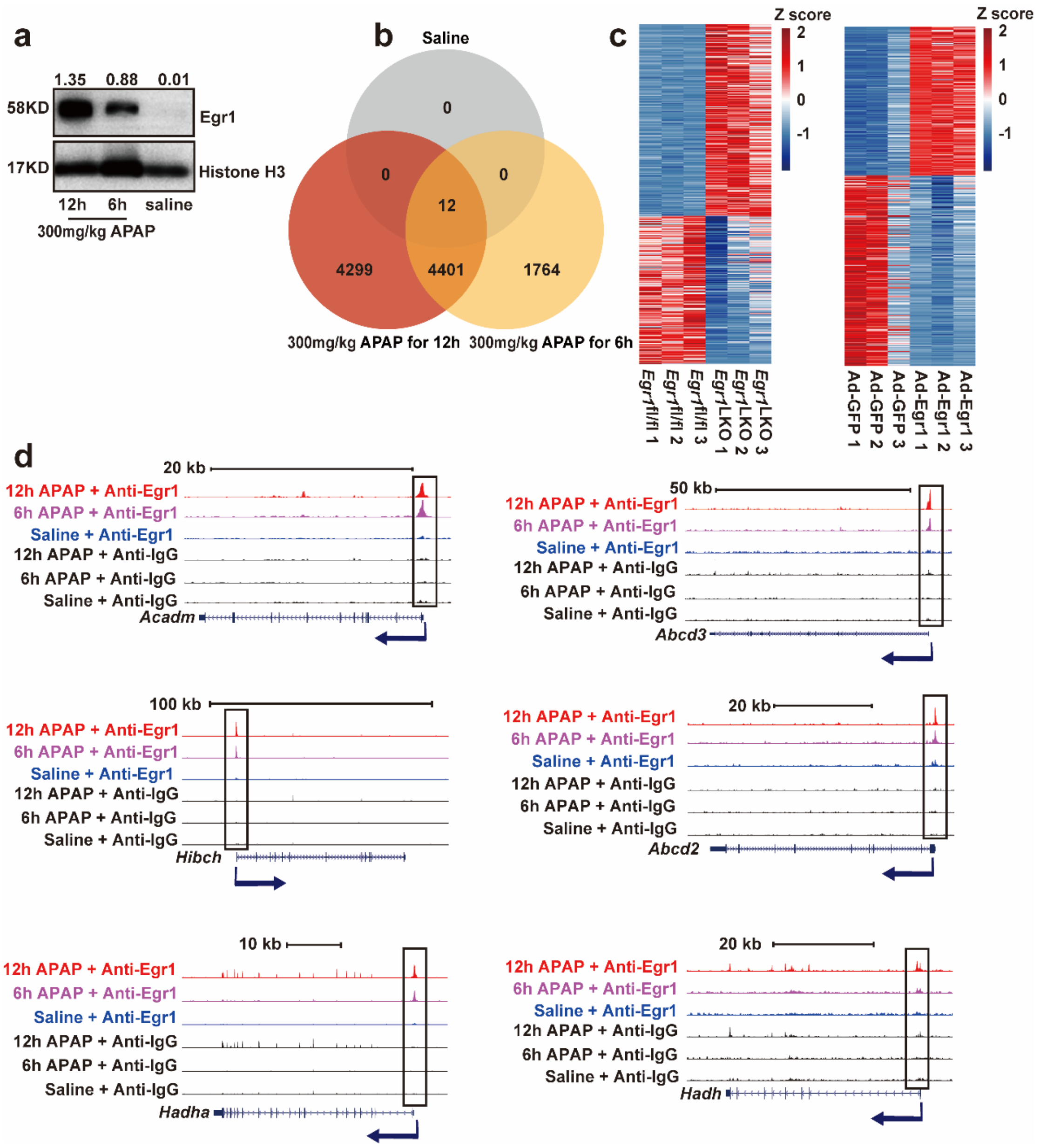

Fig. S2

a Western blot analysis of Egr1 nucleoprotein levels in the liver tissue of the mouse treated with saline for 6h, APAP for 6 h, or APAP for 12 h, the numbers above represent the quantified of the protein levels.

b Venn diagram showed the distinct peaks in liver tissue of the mouse treated with saline for 6h, APAP for 6 h, or APAP for 12 h.

c Heatmaps of the whole metabolomic profile of *Egr1*$^{fl/fl}$ AILI mice and *Egr1*^{LKO} AILI mice, and of Ad-Egr1 or Ad-GFP pretreated AILI *Egr1*^{LKO} mice.

d Genome-browser screenshots of *Acadm*, *Abcd3*, *Hibch*, *Abcd2*, *Hadha,* and *Hadh* occupancy at *Egr1* gene loci.

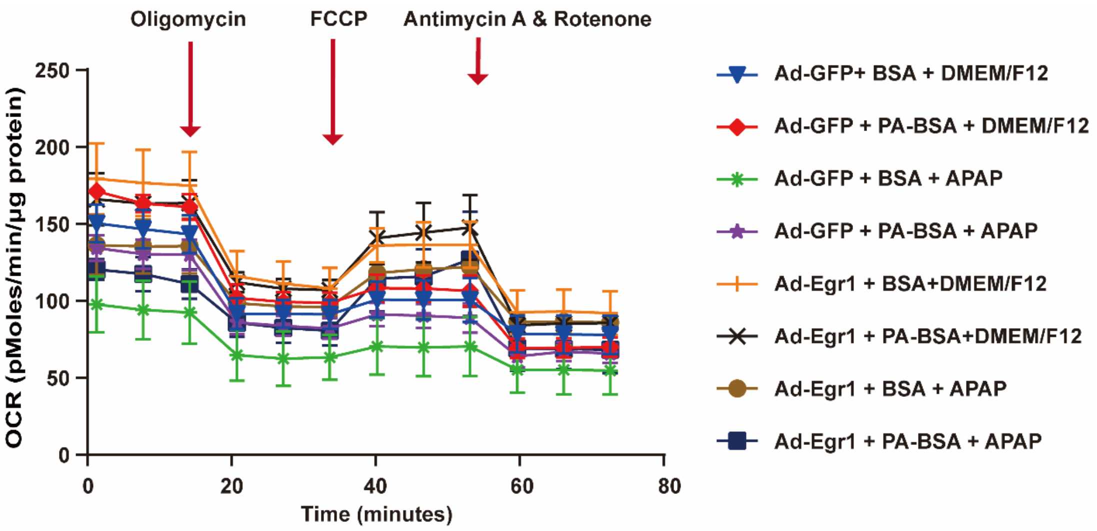

Fig. S3

AML12 cells were treated with Ad-Egr1 and Ad-CON for 48 h and then challenged with 10 mM APAP for 6 h, followed by palmitate-BSA treated for 1 h. Palmitate oxidation stress OCRs were measured using Seahorse XF96 analyzer (n = 4/group). BSA was used as a control for PA-BSA.

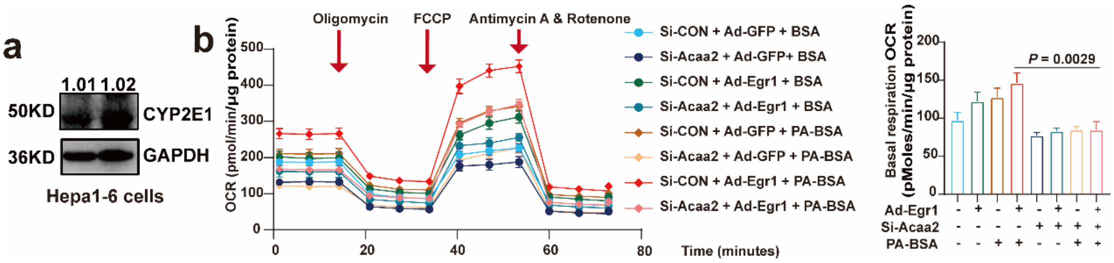

Fig. S4

a Western blot analysis of CYP2E1 levels in Hepa1-6 cells, the numbers above represent the quantified of the protein levels.

b Acaa2 was knocked down in Hepa1-6 cells at 24 h, then overexpressed Egr1 for 48 h and followed by 10 mM APAP treatment for 3 h, finally PA-BSA or BSA treated for 1 h. Palmitate oxidation stress OCRs were measured using Seahorse XF96 analyzer. Basal respiration was calculated according to instruction (n = 5–6/group, one-way ANOVA). BSA was used as a control for PA-BSA.

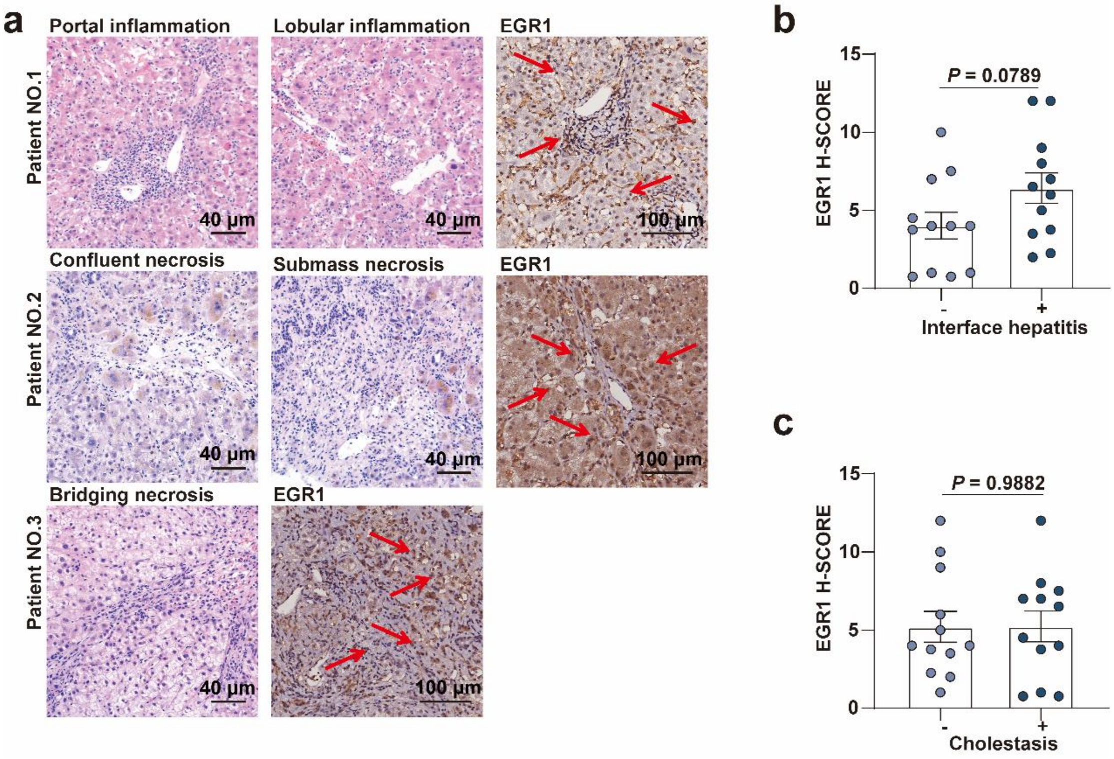

Fig. S5

a Representative images of histological features (scale bar = 40 μm) and corresponding EGR1 staining patterns (scale bar = 100 μm) in DILI patients. Red arrows indicated positive staining.

b Distribution of interface hepatitis and corresponding EGR1 H-scores in patients with DILI (*t* test).

c Distribution of cholestasis and corresponding EGR1 H-scores in patients with DILI (*t* test).
